# Health Professional Learner Attitudes and Use of Digital Learning Resources

**DOI:** 10.2196/jmir.2094

**Published:** 2013-01-16

**Authors:** Stephen Maloney, Michael Chamberlain, Shane Morrison, George Kotsanas, Jennifer L Keating, Dragan Ilic

**Affiliations:** ^1^Monash UniversityDepartment of PhysiotherapyFrankstonAustralia; ^2^Monash UniversityFaculty of Medicine, Nursing & Health SciencesClaytonAustralia; ^3^Monash UniversityDepartment of Epidemiology & Preventive Medicine, School of Public Health & Preventive Medicine, Monash University.MelbourneAustralia

**Keywords:** Information storage and retrieval, Medical Informatics, Education, professional

## Abstract

**Background:**

Web-based digital repositories allow educational resources to be accessed efficiently and conveniently from diverse geographic locations, hold a variety of resource formats, enable interactive learning, and facilitate targeted access for the user. Unlike some other learning management systems (LMS), resources can be retrieved through search engines and meta-tagged labels, and content can be streamed, which is particularly useful for multimedia resources.

**Objective:**

The aim of this study was to examine usage and user experiences of an online learning repository (Physeek) in a population of physiotherapy students. The secondary aim of this project was to examine how students prefer to access resources and which resources they find most helpful.

**Methods:**

The following data were examined using an audit of the repository server: (1) number of online resources accessed per day in 2010, (2) number of each type of resource accessed, (3) number of resources accessed during business hours (9 am to 5 pm) and outside business hours (years 1-4), (4) session length of each log-on (years 1-4), and (5) video quality (bit rate) of each video accessed. An online questionnaire and 3 focus groups assessed student feedback and self-reported experiences of Physeek.

**Results:**

Students preferred the support provided by Physeek to other sources of educational material primarily because of its efficiency. Peak usage commonly occurred at times of increased academic need (ie, examination times). Students perceived online repositories as a potential tool to support lifelong learning and health care delivery.

**Conclusions:**

The results of this study indicate that today’s health professional students welcome the benefits of online learning resources because of their convenience and usability. This represents a transition away from traditional learning styles and toward technological learning support and may indicate a growing link between social immersions in Internet-based connections and learning styles. The true potential for Web-based resources to support student learning is as yet unknown.

## Introduction

Web-based learning repositories allow mass storage, management, and search and retrieval of data for both staff and students. Material can be stored in a variety of formats and easily shared across diverse user groups. This is particularly valuable in health profession programs in which the requirement for currency demands that resources are available for regular academic review and are visible to students and their clinical educators [1,2]. Spacious electronic repositories enable a comprehensive body of resources to be accessed easily by geographically dispersed users. Repository designers can also individualize access for critical review of resources and enable ongoing resource updating and refinement [3,4]. Health profession students in workplace practice benefit from access to learning resources that promote learning experiences and maximize movement toward learning targets.

Against a tradition of hard copy learning resources [5], Internet-based learning resources are rapidly augmenting or replacing other forms of information storage and sharing [6]. Students today enjoy social immersion in Internet-based connections and embrace Internet access to learning material [7]. Students of health professions report valuing access to online resources [8,9], with students in many programs relying at least in part on Internet-based resources for learning support [2].

A 2008 systematic review examined the effects of Internet-based learning compared with either no intervention or non-Internet-based learning, (eg, classroom instruction) for students of health professions. The review identified that Internet-based learning and traditional teaching methods appeared to have similar effects with regard to student satisfaction, knowledge, behavior, and patient outcomes [6].

Little is known about the value of Internet-based resources for health profession students in supporting workplace practice: which resources they find valuable, how and when they prefer to access them, and the form of resources they prefer to access. As educators determine the resources made available to students, they may benefit from learning more about the needs and preferences of users. Packaging learning material to maximize its appeal and availability to learners can potentially increase engagement and uptake.

This study was designed to examine (1) when and why health professional learners access resources held in an online repository, (2) which resources they preferentially access, (3) what resources they find most helpful in supporting their clinical placements and development of practice competencies, and (4) whether uptake is changing across time.

## Methods

The Physeek digital repository is an online, keyword-searchable, repository of learning resources (see Multimedia Appendix for Screenshots). It was developed originally for students undertaking the 4-year undergraduate physiotherapy degree at Monash University, Melbourne, Australia. It enables remote access to learning resources to students in workplace practice. Students undertake 39 weeks of clinical learning during the third and fourth years of their studies. Physeek provides an appropriate model for Web-based learning repositories because it allows academic staff to create, store, and manage educational content in electronic format. It can be used by anyone with permission and Internet access. Third- and fourth-year students can search Physeek by using a number of search strategies (ie, subject category, keyword, year level, resource author, and resource type), whereas first- and second-year students who are not currently undertaking clinical placement can access Physeek resources through a generic learning management system (LMS), specifically Blackboard. The resources are intended to support and advance practice competency. They include lecture notes, practical demonstration videos, self-directed learning modules, and practical class pre-readings. Access to the repository was provided to clinical educators affiliated with the Monash program so that they were aware of course content and the expected level of student knowledge and skills. Educators also appreciated the opportunity to compare their own knowledge and beliefs to the current concepts taught to students.

The study was approved by Monash University Human Research Ethics Committee, approval number CF10/3439-2010001817.

### Physeek Usage Audit

Usage of the Web-based repository Physeek was audited through 2 corresponding academic semesters in 2009 and 2010. Reports for students at each year level (1-4) of the bachelor of physiotherapy degree were generated. Reports generated were (1) number of online resources accessed per day in 2010 (years 3-4); (2) number of each type of resource accessed (eg, lecture slides, practical videos, unit guides, self-directed modules, practical pre-readings [years 3-4]); (3) number of resources accessed during business hours (9 am to 5 pm) and number accessed outside business hours (years 1-4); (4) session length of each Physeek log-on (years 1-4); (5) video quality (bit rate) of each video accessed (all year levels grouped). Each video resource was loaded onto the repository in 3 different quality levels based on bit rate measured in kilobit per second (kbit/s): low quality (56 kbit/s or 150 kbit/s depending on program used), medium quality (256 kbit/s or 400 kbit/s), and high quality (512 kbit/s or 720 kbit/s). The highest bit rates allowed the greatest image resolution. Videos were uploaded onto the repository at different qualities to allow for differences in bandwidths available to students. Download time is affected by file size, and we were curious to know what file sizes students preferentially selected to download (faster downloads with poorer image resolution or slower downloads with higher resolution).

### Data Analysis

The number of online resources accessed per calendar day in 2010 were plotted against the third- and fourth-year clinical timetable.

The number of each type of resource accessed (lecture slides, practical videos, unit guides, self-directed learning modules, and practical pre-readings) in 2009 were compared to the number accessed in 2010. Chi-square tests were used to investigate if there were significant differences in uptake between 2009 and 2010 for each type of resource.

The number of resources accessed during business hours and outside business hours during 2010 were expressed as a percentage of total resource accessed for each year level (1-4). Data from first- and second-year students were included in this analysis to investigate if there were differences in the way that clinical education-based and campus-based students accessed resources. A Chi-square test was used to determine if usage within and outside business hours was significantly different.

The sum of video downloads at each quality level (low = 56 kbit/s or 150 kbit/s; medium = 256 kbit/s or 400 kbit/s; high = 512 kbit/s or 720 kbit/s) was expressed as a proportion of the total number of video downloads with associated 95% confidence interval (95% CI).

Descriptive statistics, including means, standard deviations (SD), and range were calculated to describe session length of Physeek log-ons by year level. A Kruskal-Wallis test was used to investigate differences in mean session length between each year level.

### Questionnaire

#### Participant Inclusion/Exclusion Criteria

Students enrolled in fourth year of the bachelor of physiotherapy degree (ie, those with previous clinical experience) in the year 2011 were invited to participate in the online questionnaire. Other inclusion criteria were that they were adults (>18 years), fluent in English, and had completed clinical placements in 2010. Third-year students were not included because they had not previously undertaken clinical placement and did not have experience of learning and practice in the workplace.

#### Recruitment

A bulk email invitation was sent to all fourth-year students. To reduce student perceptions of coercion, the email was sent by a research assistant who was independent of the course. The email included an explanatory statement and a hyperlink to an anonymous online questionnaire.

#### Data Collection and Analysis

The questionnaire consisted of 6 statements about Physeek usage and its impact on learning. Responses to the first 5 statements (eg, “I have found Physeek helpful in revising practical skills for clinical placement”) were graded on a 5-point Likert scale. Responses to items were assembled using descriptive statistics.

### Focus Group

#### Participant Inclusion/Exclusion Criteria

Eligible participants included fourth-year physiotherapy students (with previous clinical experience), aged over 18 years, and enrolled for study in 2011. Participants were excluded if they did not participate in clinical placement during 2010.

#### Recruitment

A convenience sample was employed to recruit eligible participants. Fourth-year students were emailed an invitation to participate by an independent research assistant. The first 18 students to reply were included in focus groups. Participants were randomly allocated to 3 groups of 6 to maximize the opportunity for individual participation [10].

#### Data Collection

Focus group discussions were facilitated by an independent researcher who was not involved with teaching in the bachelor of physiotherapy program. Group discussions were prompted by using a list of questions developed based on a review of the literature and designed to facilitate participant interaction. Each focus group was of approximately 30 minutes duration.

Questions were intended to generate general opinions about the delivery of learning resources that support workplace practice and, in particular, the usefulness of the Physeek database as a vehicle for accessing learning resources. Sessions were audiorecorded and transcribed verbatim before coding and thematic analysis.

#### Data Analysis

Transcripts for each focus group were independently coded by 2 researchers and themes were independently devised by using the principles of thematic analysis [10]. Thematic analysis indicated saturation of ideas after 3 focus groups and further participant recruitment was halted. To ensure accuracy of transcripts, a third independent researcher compared transcripts to the original audiorecording. Two researchers then compared and discussed the coded and themed transcripts for similarities and differences. This process was repeated for the transcripts of each focus group. When there was disagreement concerning major themes after 2 rounds of comparative analysis and discussion, a third independent researcher was consulted. After agreement on key themes was reached, 2 authors independently reviewed transcripts for suitable validating quotes. Quotes were also extracted in which they corroborated results of the usage audit and online questionnaire.

## Results

Participants included in the usage data were third-year students (n=62) and fourth-year students (n=57) during 2009, and third-year students (n=48) and fourth-year students (n=64) during 2010. The total number of participants over both years was 231.

### Physeek Usage Audit

#### Daily Frequency of Physeek Usage During 2010

**Figure 1:**
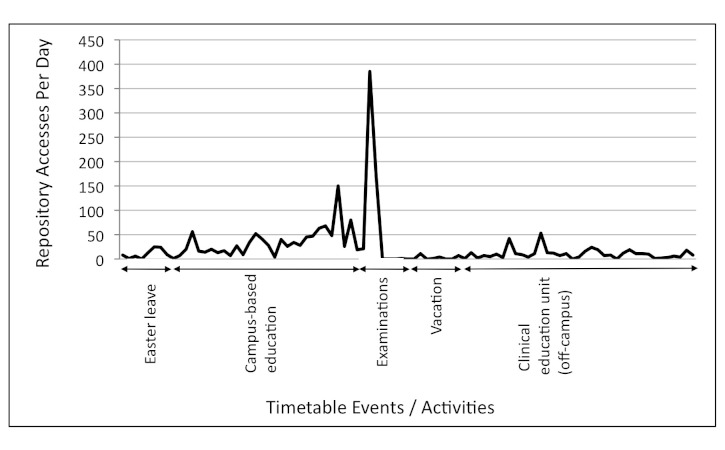
Daily Physeek usage by third-year students mapped to the semester timetable from April 1 to June 30, 2010.

**Figure 2:**
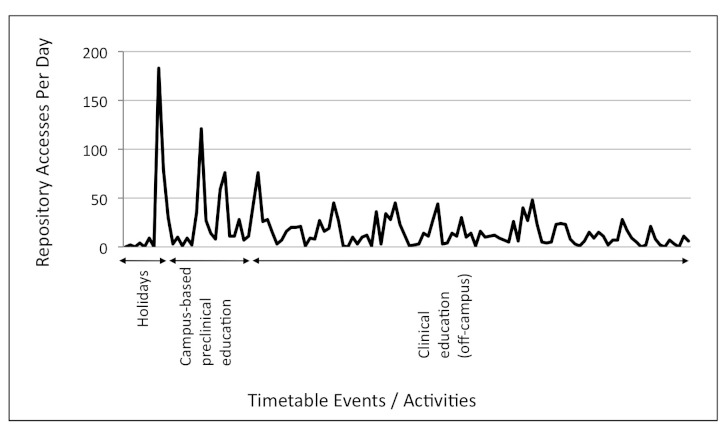
Daily Physeek usage by fourth-year students mapped to the semester timetable from February 1 to May 31, 2010.

Physeek usage by day from January 1 to September 29, 2010, is shown in Figure 1 (third-year students) and from February 1 to May 31, 2010, in Figure 2 (fourth-year students). These periods were chosen to be displayed because they represent a period of relatively intense study load and the time of highest Physeek resource uptake for each year level. Physeek usage for third-year students peaked at 385 accesses per day on May 10, 2010, at the start of the examination period, and at 183 accesses on February 8, 2010, for fourth-year students (183 accesses) immediately before the campus-based preclinical week.

#### Types of Resources Accessed From 2009 to 2010

**Figure 3:**
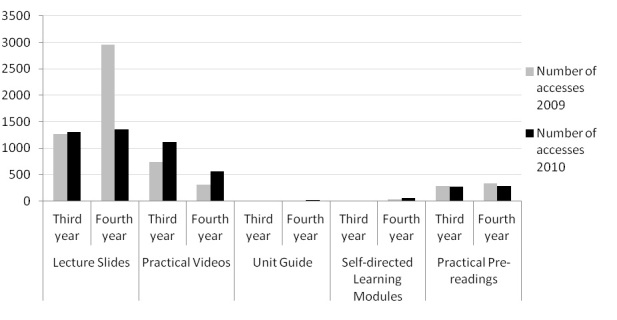
Types of online resources accessed by third-year (n=62 for 2009, n=48 for 2010) and fourth-year (n=57 for 2009, n=64 for 2010) students.

Third- and fourth-year students both accessed lecture slides and practical videos considerably more than the other resources available on Physeek. There was minimal access of unit guides and self-directed learning modules, whereas a small number of practical pre-readings were accessed (Figure 3). When use was pooled across year levels, a Chi-square test showed a significant difference in the number of lecture slides accessed in 2009 compared to 2010 (*P*=.01), with a greater number being accessed in 2009. Conversely, there were significantly more practical videos accessed in 2010 compared to 2009 (*P*=.02). There were no significant differences in uptake of unit guides (*P*=.26), self-directed learning modules (*P*=.76), and practical pre-readings (*P*=.41) between 2009 and 2010.

#### Access During Business Hours Versus Outside Business Hours by Year Level 2010

All year levels (1-4) showed a preference for accessing Physeek during business hours (9 am to 5 pm), although the proportion of access outside business hours increased with year level. First-year students accessed Physeek during business hours 68% of the time (1315 separate accesses), second-year students 59% of the time (701 accesses), third-year students 55% of the time (1815 accesses), and fourth-year students 50% of the time (1639 accesses). There was a significant (*P*=.01) difference between year levels in the amount of resources accessed during business hours and those accessed outside business hours. A post hoc analysis using Pearson correlation coefficient was performed to examine the correlation between increasing year level (1-4) and proportion of time spent using Physeek outside business hours. This returned a significant Pearson correlation coefficient of 0.98 (*P*=.02).

#### Session Length by Year Level 2010

Average session lengths for each year level ranged from 46 minutes to 59 minutes, with average session times increasing with year level (Table 1). A Kruskal-Wallis test showed that there was a significant (*P*=.01) difference in mean session length between year levels. A post hoc analysis using Pearson correlation coefficient was performed to examine the correlation between increasing year level (1-4) and session length. This returned a significant Pearson correlation coefficient of 0.99 (*P*=.01).

**Table 1:** Physeek database session length (minutes) by year level for students during 2010.

Session information	First year	Second year	Third year	Fourth year
Mean (minutes)	46.9	52.2	55.2	59.6
Number of sessions	500	284	1295	1396
Standard error of the mean	1.1	1.5	2	2.8
Median	38.6	42	35.7	38.5

#### Video Bit Rate 2010

An audit of video bit rate preference demonstrated that all available bit rates were utilized by all year levels (1-4) when accessing practical videos. Across all year levels (1-4), there were 3590 video accesses. Low quality (56 kbit/s or 150 kbit/s depending on the program) video was accessed 16% (558/3560; 95% CI 13-19) of the time, medium quality (256 kbit/s or 400 kbit/s) was accessed 45% (1614/3560; 95% CI 43-47) of the time, and high quality video (512 kbit/s or 700 kbit/s) was accessed 39% (1388/3560; 95% CI 36-41) of the time.

### Online Questionnaire

Thirty-nine students completed the online survey (81% response rate). A summary of student responses is presented in Table 2.

**Table 2:** Likert scale responses to survey regarding Physeek utilization.

Question	Response, n (%)^a^				
	Strongly disagree	Disagree	Neutral	Agree	Strongly agree
I have found Physeek useful in revising practical skills for clinical placement	1 (2.6)	0 (0)	0 (0)	18 (46.2)	*20 (51.3)*
Physeek has increased the efficiency of searching for and locating clinical revision resources	1 (2.6)	0 (0)	0 (0)	10 (25.6)	*28 (71.8)*
Physeek has increased the time I have spent revising clinical skills	0 (0)	2 (5.1)	12 (30.8)	*15 (38.5)*	10 (25.6)
Physeek has increased the likelihood that I would seek a resource before its intended delivery	0 (0)	4 (10.5)	5 (13.2)	*18 (47.4)*	11 (28.9)
Access to resources via Physeek has enhanced my clinical performance	1 (2.6)	0 (0)	6 (15.4)	*21 (53.8)*	11 (28.2)
I have chosen not to attend a lecture or practical session in the past 12 months due to a knowledge that resources, ie, lecture notes, would be available on Physeek^b^	*20 (51.3)*	13 (33.3)	4 (10.3)	2 (5.1)	0 (0)

^a^ Mode response for each question is represented in italics.

^b^ Responses for this question correspond to never, rarely, sometimes, frequently, and always instead of strongly disagree, disagree, neutral, agree, and strongly agree, respectively.

### Focus Groups

There was a general consensus among participants that the Physeek database provided an easier and more efficient means of accessing resources than traditional methods of delivery. Most students agreed that this provides an advantage in supporting clinical performance. However, student experiences varied with regard to the ways they utilized the repository, For example, a number of students viewed Physeek primarily as a “quick access” revision tool, but many also saw the potential for an online repository to be the primary mode of delivery for new or additional material. Similarly, students were divided on the future incorporation of technology and electronic education into health care practice. Results are presented for 3 key themes: (1) an online searchable repository is the preferred method of accessing learning resources efficiently in workplace practice; (2) make it bigger, faster, and easier; and (3) online repositories may be an effective tool to support lifelong learning and health care delivery. Supportive quotes demonstrating each of these themes are provided.

#### An Online Repository Is the Preferred Method for Efficient Access to Learning Resources

A digital repository such as Physeek cannot have a positive effect on learning and physiotherapy practice if students do not access its resources. Students varied somewhat in the extent to which they felt Physeek was useful to their clinical performance, but all agreed that Physeek was the easiest way to access resources needed for clinical revision. This comment from one student encapsulated the feelings of most participants when comparing Physeek to other means of accessing resources to support clinical education:

...you can’t even compare them...there’s just that instant access point [to the learning resources via Physeek] when you’re on clinic.

There were a number of reasons why students preferred an online repository as opposed to other methods. For example, one topic that presented several times was the idea of efficient access to relevant resources:

Physeek’s been really useful because you don’t have to go and sift through...

If...I didn’t know where to look for a piece of information, I could just search it and it would come up with a list of things that could possibly be useful.

Not only did students feel that Physeek was an efficient way of accessing resources, they also believed that the resources could be accessed at a time that was clinically relevant:

...especially on clinics when I was doing outpatients, we’d know our patient the day before so then I’d go home that night and look at all the tests and everything that I could do, and think about all the treatment options...

One of the other advantages of having an online repository raised by participants was linked to the idea of resource reliability and academic review. Many students felt that because resources were produced and updated by an academic organization (ie, the university, evidence-based resources), they were more confident in using them:

You know it’s reliable instead of like Google, at least stuff on Physeek is actually correct.

#### Students Want a Repository That is Larger, Faster, and Easier to Use

The focus groups revealed a number of student recommendations for how an online repository might provide resources more effectively to facilitate clinical performance. These recommendations primarily focused on the idea that Physeek could be expanded, while improving the search functionality of the repository. Some students remarked that, at present, they had some difficulty in finding relevant resources using the Physeek search function:

...there are other times when I’ve just searched for something and the wrong information comes up.

Several students suggested that mapping or browsing functions may make searching the database more accurate, while increasing the number of resources relevant returned for consideration:

If you typed “neuro” [neurological] it would...map it to everything that’s related to neuro so say I had a neuro placement coming up, you could go, I don’t know what I want to learn, but I’ll just...search and see what resources they’ve got.

Students also felt that a browse function may enhance their ability to search through resources, enabling them to look for cues regarding knowledge and skills that may be useful:

...you get a browse function so...you could just go look at musc [musculoskeletal] and just scroll through every musc thing we’ve done.

Lack of technological prowess did not seem to affect opinions about Physeek. One of the key factors in facilitating uptake of Physeek resources may be related to limiting the technological knowledge and skills needed to use the database:

No, I hate technology and that’s why I like Physeek, because it’s just like Google. If I don’t know what I’m looking for specifically, I can just type in MS [multiple sclerosis] and everything will come up.

#### Online Repositories May Be an Effective Tool to Support Lifelong Learning and Health Care Delivery

There are potential advantages to integrating technology into health care practice. Students’ attitudes toward lifelong learning and the capacity for an online repository to enhance this demonstrated the capacity for an online repository to improve individual health care delivery:

It would help if we still had access to it after we finish...because as a new grad, you’re still going to be wanting to revise.

These attitudes reflect a desire by the students to continue learning and advancing their profession after graduation:

If you don’t embrace it, physio’s just going to be left behind and everyone’s just going to be doing what we did twenty, thirty years ago, nothing’s going to change.

## Discussion

This paper presents the results of a multifaceted study examining student usage and perceptions of a Web-based digital repository designed to improve access to learning resources for health professional learners. This is the first study to examine students’ usage of an online learning repository as a support for workplace practice and relate this to student experiences and preferences for learning resources. The findings of this study provide insight into how online repositories could be designed or utilized by educational institutions to enable maximal resource access and uptake for health professional learners.

Students saw the Web-based repository as the most efficient and the preferred source of learning resources, but also felt that Physeek could be further improved to make resource access even more efficient. This emphasis on efficiency is reinforced by comments that a major advantage of a Web-based repository is a reduction in the need to wade through irrelevant search yields. This may account for the rapid uptake of Physeek resources. When one finds useful information in a time-efficient way, this is likely to provide positive reinforcement to the exercise of looking for relevant material. This feedback loop encourages students to become active learners.

The results of the online questionnaire and usage audit suggest that Physeek may have had a positive impact on student study habits. Students reported that Physeek had increased the time they spent on clinical revision, and the likelihood that they would seek out resources before their intended use, again indicating that a Web-based repository may have made students more willing to engage in independent learning.

An audit of Physeek usage found that students were most likely to access online learning resources to satisfy immediate academic requirements, such as examination preparation or for gathering information needed on clinical placements. Physeek usage for both third- and fourth-year students peaked at times of increased study load (eg, third-year examinations and the fourth-year preclinical preparation period). These results are encouraging because they demonstrate a willingness by students to revise learned knowledge by using Web-based resources.

With the immediate access to knowledge and skills that an online repository provides, there is the potential for students to feel that they can rely on the repository as a source of information at the point of service delivery. All year levels in 2010 accessed resources through Physeek during business hours (9 am to 5 pm) significantly more than outside business hours. However, the first 2 years of study are primarily campus-based, allowing considerable time for computer access on campus during business hours, whereas both third- and fourth-year students accessed resources outside business hours more than 45% of the time while on placement. Revision session length for third- and fourth-year students was also typically greater than 55 minutes when logged on to Physeek.

This represents a considerable amount of time spent revising material on Physeek outside of clinical placement hours. Key student motivators for after-hours access are not clear, and may have been influenced by a lack of time during clinical hours, reduced Internet access, or because the use of mobile devices at the point of care is currently discouraged in most clinical environments. Another study limitation is that although there is no motivation for accessing the repository without utilizing its resources, behavior of this kind would impact the accuracy of the usage data.

It was clear that the availability of resources through a Web-based repository did not appear to have a negative impact on attendance at scheduled learning sessions. More than half (51%) of the students in the study reported they had never skipped a lecture or practical session in the past 12 months because the learning resources were also available on Physeek. Another 33% said that they rarely did so. Only 2 students (5%) said that they missed sessions frequently because the resources were available on Physeek. Students reported that they were more likely to use Physeek to enhance or broaden their knowledge rather than as an alternative to lectures or practical classes. This supports the findings of an observational study by Grabe and Christopherson [2], who found that providing supplementary online resources, in this case lecture notes, could be related to improved class attendance. In this study, an online repository appears to enable meaningful revision and consolidation of knowledge, as well as immediacy of information at the point of application. A repository such as Physeek may, therefore, enhance clinical learning and practice, as well as patient care.

Students overwhelmingly preferred lecture notes and practical videos as the learning resources they accessed online. This is in agreement with the focus group discussions in which students were almost unanimous in reporting that these were the resources most helpful to them. It may be students view these as critical information for professional performance, whereas self-directed learning modules and practical notes are outside the core curriculum. However, one group of students appeared not to realize that these non-core resources were available on Physeek. Because Physeek provides only a search function and not the capacity to browse topics, it is possible that students were not aware of all the resources available to them via the online repository. As suggested by a number of students in the focus groups, a browsing or cataloging system may overcome this limitation and increase uptake of different types of resources.

Students tended to prefer better quality video for practical skill revision, with the majority of videos accessed of medium or high quality. However, approximately one-fifth of video accesses in 2010 (n=588) utilized low quality video. It was suggested from the focus group transcripts that this may be a matter of convenience. For example, students who needed to access video quickly or at slow connection speeds might use lower bit rate video at one time, and preferentially utilize higher quality video when they had access to greater Internet bandwidth. A qualitative study by Blake [9] also found that it is this type of flexibility that attracts students to the use of online material. This supports one of the major themes from the focus group, of making the repository “larger, faster, and easier to use.” Students generally wanted more resources available through Physeek, while at the same time maintaining or improving the ease and efficiency of finding these resources.

As yet, no major modifications have been made to the repository; therefore, it appears that the uptake of Physeek resource access was primarily driven by growing awareness and familiarity with the repository, and an increasing student desire to access resources online. The results of Chi-square tests comparing uptake of each resource between 2009 and 2010 indicated that usage remained consistent over this time. Given that the technology for Physeek was implemented at the start of 2009 and students were still familiarizing themselves with using a Web-based repository, the significant amount of time spent on Physeek over the 2-year period (Table 1) is encouraging. These data will need to be re-evaluated as modifications are made to the repository based on student feedback. Further research could also be conducted to identify which aspects of the Physeek repositories’ properties and functionality has influenced its successful implementation, such as the particular tagging schema utilized allowing resources to be searched by category as well as per individual item, the consistent format of video resources allowing playback by the built-in media player, or the ability for other learning systems to link to resources within the Physeek repository.

Rather than providing only supplementary material, it is possible that Web-based repositories can be the primary means of accessing resources for health professional learners, as well as a tool for educating patients and accessing “practical” information. For example, Physeek may be useful in providing patient education materials that can be readily accessed at the bedside. When asked about possible further applications of the online repository, students pointed to the capacity to improve clinician recall and patient education by using interactive media, while also enhancing lifelong learning for health care professionals.

The results of this study indicate that today’s health professional students welcome the benefits of online learning resources because of their convenience and usability. This represents a transition away from traditional learning styles toward technological learning support, and may indicate a growing link between social immersion in Internet-based connections and learning styles. The true potential for Web-based resources to support student learning is as yet unknown. Many students expressed a desire to continue using this type of resource throughout their professional lives. With careful design, a Web-based learning repository is potentially a useful model to provide support for health professional students and graduates in workplace practice.

## Abbreviations

kbit/s: kilobit per second
LMS: learning management system

## Multimedia Appendix 1

Screenshot 1 - Example of the repository search yield display.

## Multimedia Appendix 2

Screenshot 2 - Example of a repository item description.
