# Supplementary figures and images for: Health Professional Learner Attitudes and Use of Digital Learning Resources
Source: J Med Internet Res. 2013 Jan 16;15(1):e7. doi: 10.2196/jmir.2094 (PMC3636135; doi:10.2196/jmir.2094)

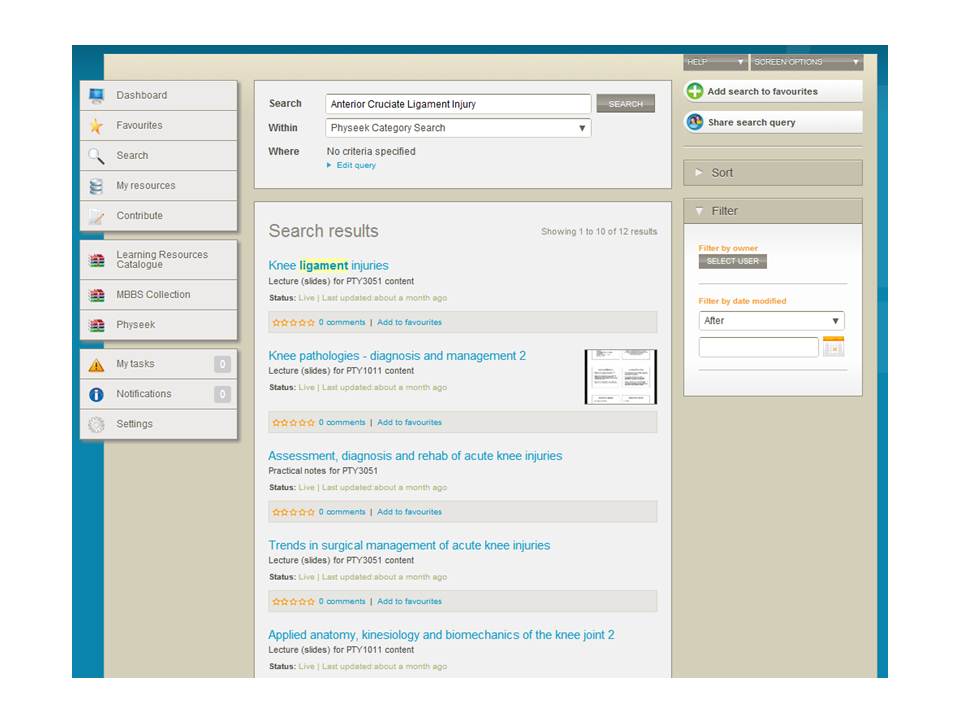

Supplement: Supplementary file 1 [file jmir_v15i1e7_app1.JPG]

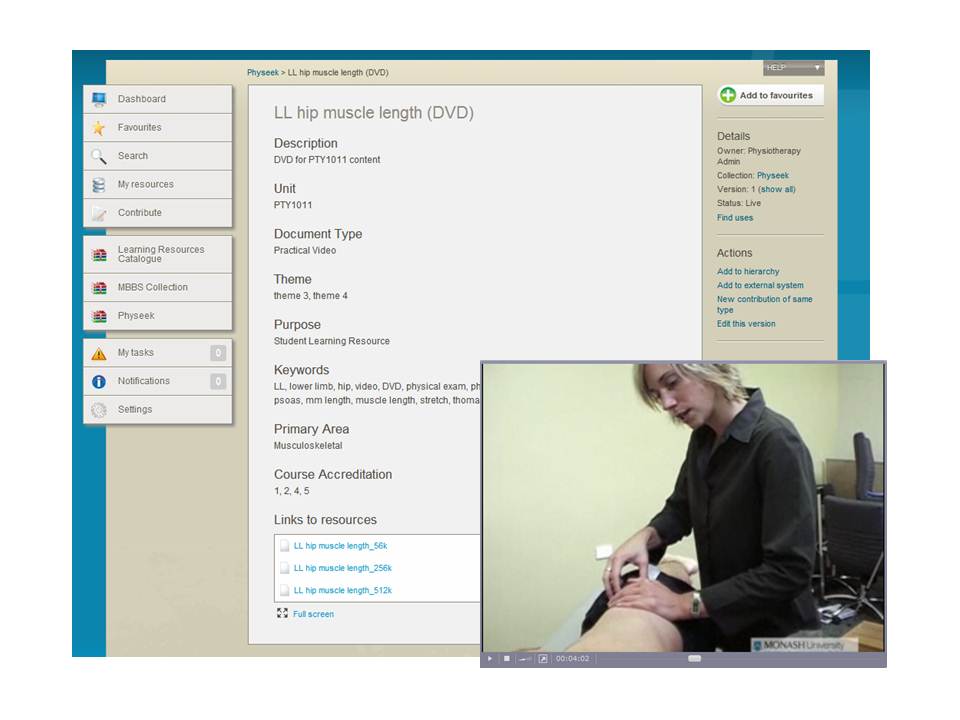

Supplement: Supplementary file 2 [file jmir_v15i1e7_app2.JPG]
